# 

**Published:** 1891-12

**Authors:** 


### CONTENTS:

Article I.—Examining Boards *vs.* Dental Colleges. By Dr. W. C. Barrett. - - - - - - - - - 337

" II.—Antral Abscess as a Result of Alveolar Abscess. By Dr. M. O. Cooley. - - - - - - - 356

" III.—The Missing or Suppressed Teeth in Man. Inaugural Address. By Andrew Wilson, Esq., L. D. S. - 362

" IV.—Gold as a Material for Filling Teeth. R. E. Sparks, L. D. S. - - - - - - - - - - 368

" V.—Corrosive Sublimate as a Disinfectant Against the Staphylococcus Pyogenes Aureus. - - - - 371

" VI.—Cocaine. By F. A. Stevenson, D. M. D., L. D. S. - 374

### BIBLIOGRAPHICAL.

Dental Medicine. By F J. S. Gorgas, A. M., M. D., D. D. S. - 377

### MONTHLY SUMMARY.

A Case of Cocaine Poisoning. - - - - - - - 379

Capping Pulps. - - - - - - - - - - 381

Fitting Bands. - - - - - - - - - - 382

The Physiology of Digestion in Infants. - - - - 383

Gorgas Dental Medicine, Fourth Edition. - - - - 384

Keep Clean. - - - - - - - - - - - 384

